# Can Brief Listening to Mozart’s Music Improve Visual Working Memory? An Update on the Role of Cognitive and Emotional Factors

**DOI:** 10.3390/jintelligence12060054

**Published:** 2024-05-23

**Authors:** Vaitsa Giannouli, Juliana Yordanova, Vasil Kolev

**Affiliations:** 1 Institute of Neurobiology, Bulgarian Academy of Sciences, 1113 Sofia, Bulgaria; jyord@bio.bas.bg (J.Y.); kolev@bio.bas.bg (V.K.); 2 School of Medicine, Aristotle University of Thessaloniki, 54124 Thessaloniki, Greece

**Keywords:** Mozart K448 sonata, preferences, positive and negative affect, visual working memory

## Abstract

The aim of this research was to enhance understanding of the relationship between brief music listening and working memory (WM) functions. The study extends a previous large-scale experiment in which the effects of brief exposure to music on verbal WM were explored. In the present second phase of the experiment, these effects were assessed for the visuospatial subcomponent of WM. For that aim, visuospatial WM was measured using the Corsi blocks task-backwards and Visual Patterns Test in a large sample of 311 young and older adults after being exposed to musical excerpts coming from different music composers (Mozart, Vivaldi, Glass). To account for possible effects of arousal, a silence condition was used. Individual preference for music excerpts and emotional reactions to each condition were also subjectively rated using the Positive and Negative Affect Schedule (PANAS) to account for the role of emotional reactions in shaping subsequent cognitive performance. Results showed that music affected the visuospatial sketchpad of WM. In line with the previously described Mozart effect, only Mozart’s music had a significant positive impact on visuospatial WM in the two age groups, regardless of preferences, and on overall age-related WM decline in older adults. The Mozart effect was more prominent for the VPT than the Corsi task and was also expressed for the prevailing positive effect. These observations are in contrast to the selective influence of Vivaldi’s music on verbal WM that was detected in our first study. Together, the results demonstrate a differential music influence on the phonological loop and on the visuospatial sketchpad. They thus contribute to the debate of whether music has the potential to affect distinct processes within working memory in an excerpt- or composer-specific manner. Also, they suggest that emotional activation and central executive attention are essentially involved in modulating the influence of music on subsequent cognition. These findings can assist in the selection of music excerpts used in cognitive rehabilitation programs that focus on visuospatial skills.

## 1. Introduction

The effect of brief exposure to music on cognitive performance has been broadly recognized by the so-called ‘Mozart effect’ (Rauscher and Shaw 1998). The Mozart effect refers to a “slight and transient improvement in spatial [*sic*] reasoning skills detected in normal subjects as a result of exposure to the music of Mozart, specifically his sonata for two pianos (K448)” (Pryse-Phillips 2003), and was first described in 1993 (Rauscher et al. 1993).

Accordingly, early investigations of the Mozart effect primarily focused on visuospatial capacity. The majority of studies followed the procedure originally introduced by Rauscher et al. (1993), employing mainly the Paper Folding and Cutting tasks (Črnčec et al. 2006; Carstens et al. 1995; Gilleta et al. 2003; Lin and Hsieh 2011; Lints and Gadbois 2003; McCutcheon 2000; McKelvie and Low 2002; Newman et al. 1995; Steele et al. 1999; Stough et al. 1994; Wilson and Brown 1997). However, the conclusions remained inconclusive (Giannouli et al. 2017; Giannouli and Popa 2017) and limited to the visuospatial capacity. Importantly, later studies have found that music experience may prime subsequent cognitive processing in other domains such as language (Koelsch et al. 2002; Patel 2003; Ettlinger et al. 2011), memory (Giannouli et al. 2010) and attention (Giannouli 2012; Moreno and Bidelman 2014).

To account for the immediate effects of music experience on a broader range of cognitive functions, working memory (WM) has been considered (Mammarella et al. 2007). The term working memory (WM) refers to a limited-capacity memory system that is responsible for storage and manipulation over short periods of time (Baddeley 2000). It is related to a variety of cognitive abilities, including processing speed (Conway et al. 2002), spatial ability (Miyake et al. 2001), and verbal abilities like language learning (Baddeley et al. 1998b), reading (De Jong 1998), and selective attention (Baddeley et al. 1998a). According to the model of Baddeley (2000), WM consists of one attentional control system, *the central executive*, and three supplementary systems: the *visuospatial* sketchpad responsible for holding and operating visual input (Della Salla et al. 1997), the *phonological* loop dealing with speech-based information (Papagno et al. 1991), and the *episodic buffer* unifying visual, spatial, and verbal information into articulated units according to their temporal order. An important research question in relation to the extended cognitive effects of music was whether musical experience might influence different sub-components of WM tackled separately or jointly (Giannouli et al. 2019).

In this perspective, our previous study (Giannouli et al. 2019) explored whether brief exposure to music might modulate subsequent processing of linguistic material in a large sample of healthy participants. Two verbal cognitive functions were targeted: verbal working memory and phonologically cued semantic retrieval from long-term memory, as reflected by the forward digit span test (F-DST) and word fluency test (WFT), respectively. It was found that brief exposure to music pieces from different composers (Mozart, Vivaldi, and Glass) had no beneficial effect on verbal WM, with even a transient impairment emerging after Vivaldi. In contrast, Vivaldi’s excerpt induced a marked enhancement of word fluency. Likewise, Mammarella et al. (2007) have reported that brief exposure to Vivaldi’s music affects the capacity of phonological working memory. These previous results show that listening to music can facilitate or inhibit verbal functions, providing evidence for the possible selective effect of Vivaldi’s music on the phonological sub-component of WM (Giannouli et al. 2019). Together with the original findings on the effect of Mozart’s music on the visuospatial sub-component of WM (e.g., Rauscher et al. 1993), they imply that depending on composer- or excerpt-specific music features, specific sub-components of WM can be selectively affected.

One possible explanation for such specificity, for example, is that both music and language faculties are supported by a distinct neural system for processing structured auditory regularities (Strait et al. 2011). Koelsch et al. (2002) have demonstrated that processing chord sequences activates a cortical network identified as domain-specific for language processing. Comparative neuroimaging and electro-magnetoencephalographic studies have further confirmed that discrete structured elements arranged in rhythmic sequences characterizing both music and language are processed by a common neural system in the brain (Patel 2003; Koelsch et al. 2013), largely engaging the neural structures of implicit memory (Ettlinger et al. 2011; Ullman 2001), in contrast to explicit attentional control in working memory. In the same vein, it has been demonstrated that listening to music activates those brain regions in the prefrontal, temporal, and precuneus cortex that are engaged in visuospatial processing and may serve to prime and facilitate information processing in these regions during spatial reasoning tasks (Jenkins 2001).

On the other hand, there are hypotheses according to which listening to music does not modulate specific processing circuits but instead influences performance via unspecific activations. The critical point in these theories is that listening to music affects arousal (degree of physiological activation), emotional activation (short-lived physiological reactions, e.g., enjoyment), and mood (long-lasting emotional tone), which in turn may influence performance on a variety of cognitive tasks. The inverted U-shaped effect of arousal on performance has since long been recognized (e.g., Baldi and Bucherelli 2005). By demonstrating that there is no difference in the effects of music and traffic sounds on performance, Roth and Smith (2008) have explained such observations in terms of physiological reactions evoked by heightened arousal and introduced the *arousal framework*. Likewise, according to the *arousal-and-mood* hypothesis of Thompson et al. (2001), listening to music affects cognitive performance by shifting emotional arousal and activations. Emotions have a particularly strong influence by modulating the selectivity of attention and motivation. According to the *broaden-and-build theory* (Fredrickson 1998, 2001), positive emotions broaden the scopes of attention, cognition, and actions, and phenomenologically distinct positive emotions can produce a similar broadening relative to neutral states. On the contrary, negative emotions shrink these domains (Fredrickson 1998, 2001). In a similar vein, the *attentional control theory* (Eysenck et al. 2007) posits that a heightened activation/emotional state (e.g., anxiety) impairs the efficient functioning of the goal-directed attentional system and increases influences by the stimulus-driven attentional system, leading to an imbalance in executive attentional control. In view of these concepts, the influence of music may primarily tackle the central executive sub-component of WM through unspecific modulations of attention after arousal and emotional transitions rather than through specific recruitment of the phonological or visuospatial loops. Currently, the mechanisms mediating the effects of music on cognitive performance are not fully understood.

In this regard, the major objective of the present study was to further highlight the involvement of the sub-components of WM in shaping cognitive processes after a brief musical experience. For that aim, we used the musical excerpts of Mozart, Vivaldi, and Glass as in our initial study, where the phonological loop was targeted (Giannouli et al. 2019) in order to assess *visual spatial performance* after music. We used the same examinees and the same musical excerpts in order to explore whether music would affect similarly or differentially the visuospatial WM. This approach was chosen to address the specificity of involvement and responsiveness of WM sub-systems, given also that background music has been found to affect to a greater extent visuospatial as compared to verbal memory (Echaide et al. 2019).

In the present study, to explore the visuospatial WM sub-system, we used two tests: the Corsi block tapping test (Corsi 1972; Kessels et al. 2008) and the Visual Patterns Test (VPT; Della Salla et al. 1997). These two tests are acknowledged instruments for assessing the visuospatial WM. However, they differ in the extent to which they engage non-visual executive processing because, in contrast to the VPT, the Corsi test requires remembering both the spatial location and sequence of visual information, thus entailing central executive mechanisms (Brown et al. 2006). The two tests may also tackle, to a different extent, the phonological/semantic sub-components of WM. In visuospatial tasks, there may be an interaction between verbal and visual WM components since verbal coding may influence visual matrix task performance (Brown et al. 2006). It has been demonstrated that the visual patterns in the VPT are difficult to verbalize, which limits the engagement of the phonological WM loop (Nicholls and English 2020). Thus, by minimizing the contribution of central executive and phonological/semantic WM sub-components, the VPT is considered a more direct measure of visuospatial WM. In the context of the present study, it is important to emphasize that although the visual WM is associated with a plethora of neuropsychological tests measuring processing speed and executive function, it is not correlated with verbal fluency (Brown et al. 2012), which was specifically sensitive to music excerpts in our previous study (Giannouli et al. 2019).

To further distinguish the role of WM sub-components in mediating visuospatial performance after music, we studied groups of young and older healthy adults, as included in the original study. It has been consistently reported that executive control, working memory, and attentional functions decline with aging (Grady 1998; Reuter-Lorenz 2002; Grivol and Hage 2011). Specifically, visual WM is age-sensitive during standard cognitive testing, with a demonstrated overall decline in measures as age increases (Brown et al. 2012; Nicholls and English 2020). In particular, specific forms of spatial-sequential WM tasks such as the backward spatial span are significantly predicted by age (Brown 2016), but no research so far has focused on the marked effects of aging on visual WM when interventions (even passive ones) such as listening to music excerpts are applied. Also, the intentional use of visual or verbal processing strategies is age-dependent, with young adults reporting more verbal strategies for visual tasks (Nicholls and English 2020). Hence, we hypothesized that if musical experience affects visuospatial cognition via cognitive control mechanisms, the outcome would differ between young and older participants. Also, the outcome might additionally depend on the task, the Corsi test or the VPT, due to age-dependent differences in both attentional and verbal strategies. Whereas long-lasting positive effects of musical training in aging are well documented (e.g., Bidelman and Alain 2015; Moussard et al. 2016), it has not been clarified so far if brief exposure to music may produce fast modulations of visuospatial processing in individuals with declining WM capacity. Addressing this question is relevant for practical and therapeutic applications with older people.

As in our original study (Giannouli et al. 2019), arousal and emotional activations were accounted for as possible mediators of cognitive performance after brief exposure to music. First, familiarity with the excerpts was an exclusion criterion because it is not clear from previous similar research whether any positive or negative influences are derived from the emotional involvement typically induced by familiar music (Mammarella et al. 2007). Second, exposure to silence was a condition that was introduced to be contrasted with multiple excerpts and to serve as a control for the effects of arousal. In addition, to account precisely for emotional activations, (1) online preference to different music excerpts was scored, and (2) the immediate emotional reaction to each music condition (Mozart, Vivaldi, Glass) and silence was measured using the Positive and Negative Affect Schedule (PANAS; Watson and Clark 1999). It is interesting to note that within the ‘Mozart effect’ research tradition, emotional modulations play a major role in modifying visuospatial performance after music, as formulated in the arousal-and-mood hypothesis of Thompson et al. (2001). More recent reports support this notion by demonstrating that the induced emotions imposed by different music conditions critically influence visuospatial performance on tasks such as Corsi (Palmiero et al. 2016). Ribeiro et al. (2023) also find that music evoking positive emotions boosts visuospatial WM performance in young adults.

In considering all these open issues, the specific questions addressed in the current study were: (1) Do music excerpts of Mozart, Vivaldi, and Glass influence the visuospatial sub-component of WM? (2) Is there a differential effect of music on visual WM according to the age (young and older) of participants? (3) May personal music preferences, condition-induced emotions, and/or music excerpt characteristics differentiate participants’ visual WM performance after exposure to music?

## 2. Method

### 2.1. Participants

We approached the participants previously examined in the first phase of this large-scale experiment in order to re-examine them in a follow-up study focused on another cognitive domain. From the initial 448 individuals, 311 agreed to be re-examined. Although attrition was present (22.25% of the initial sample of participants were not re-examined), this was not due to withdrawal of consent, a change in the eligibility of the participants (health status), and/or a decline to be re-examined. The reasons were that some had changed home addresses and telephone numbers, while some older adults (N = 14) had died, thus rendering a second examination impossible. Sample size estimation was performed with the G*power software (Release 3.1.9, Faul et al. 2009). A priori G*Power analysis suggested a total sample size of 132 participants for a repeated measurement design, an alpha error probability of 0.05, a desired power of 0.80, two groups, and four measurements. For the present study, the re-recruited 311 participants exceeded the recommended sample size.

A total of 159 young adults (90 women, 69 men; mean age = 28.77 years, SD = 8.83; mean duration of education = 14.35 years, SD = 1.39) and 152 older adults (93 women, 59 men; mean age = 71.92 years, SD = 6.84; mean duration of education = 7.65 years, SD = 3.79) participated in the experiment. As in the first study, the re-recruited subjects were re-examined for suitability for participation using the same criteria. The inclusion criteria for young and older adults were: no past or current psychiatric diagnosis and no cognitive deficits as measured with a score over 27 points in the Mini Mental State Examination (MMSE) or the equivalent Hindi Mental State Examination (HINDI) (for the participants with less than three years of education). Some older adults had been taking medication related to cardiovascular diseases. None of them had an official diagnosis of a cognitive deficit, and all had scored above 27 points on the Greek version of the MMSE or the equivalent HINDI, thus excluding dementia. The exclusion criteria for both the young and older participants were a history of psychiatric or neurological disorders or substance abuse dependence, a history of head injury, or any other medical condition (including significant perceptual deficits such as visual and/or hearing impairments not corrected sufficiently by aids) that might affect neuropsychological performance. Non-native Greek language speakers were also excluded. All participants were non-musicians, and the current occupation of young adults as well as the previous occupation of retired older adults were not related to music.

### 2.2. Musical Stimulation Material 

The volunteers participated in four listening conditions. Each condition lasted for approximately 10 min. The music excerpts were: (1) Mozart’s sonata for two pianos in D major (Allegro con spirit K.448); (2) Vivaldi’s harpsichord concerto Op. IV n.10;(3) Glass’s Music With Changing Parts; and (4) a silence condition without any acoustic stimulation.

### 2.3. Visual Storage in Working Memory

**Visual Patterns Test:** A validated Greek version of the Visual Patterns Test (VPT) was administered as suggested by Della Salla et al. (1997). The participants were presented with cards displaying combinations of checkerboard patterns constructed in such a way that it was not easy to encode the patterns verbally. Each visual pattern was produced by having half of the squares in a presented grid filled in, so that some of the squares were black and the others white. As described in Della Salla et al. (1997), the grids progressed in size from the smallest, a 2 × 2 matrix (with two filled cells), to the largest, a 5 × 6 matrix (with 15 filled cells). Complexity was steadily increased by adding two more cells to the previous grid. The level of complexity of a pattern is defined as the number of filled cells in a grid and thus ranges from a minimum of 2 (for the 2 × 2 matrix) to a maximum of 15 (for the 5 × 6 matrix). In each experimental set, there were three patterns at each level of complexity, carried in three grids of the same size and shape. Each grid was presented to participants for 3 s. During that period, participants were asked to memorize the cards and to respond immediately after the presentation by reproducing the black squares they saw in the presented cards, indicating with pencil the answer on an empty sheet grid of the same size. The test was interrupted after 1 error, not after 3 consecutive errors at the same level of difficulty as in the original VPT. The final score was in the range of 2–15, reflecting the number of all the correct squares that were drawn as black before an incorrect response was given. The suggested parallel versions A and B (Della Salla et al. 1997, 1999) were implemented in a randomized way.

The exact instructions to the participants were as follows: “This task tests the memory for visual images. You are going to see a pattern like this one (showing a stimulus card) and I will ask you to recall it by drawing in these empty grids (showing the answering sheet). You are going to look at the pattern for a short period of time. For that reason you should concentrate and look carefully in order to recall the pattern immediately afterwards. As soon as I cover the pattern you should start drawing. The patterns are easier in the beginning and become more difficult later on.” After each music condition, different sequences with equal complexity were presented to the participants in a way that was similar to the process of adding one item for each testing session for the Forward Digit Span Test.

### 2.4. Visual Storage and Processing in Working Memory

**Corsi blocks task backwards condition:** The Corsi block tapping test assesses visuospatial WM (Corsi 1972; Kessels et al. 2008). It involves mimicking a researcher as he/she taps a sequence of up to nine identical spatially separated cubes, unevenly distributed on a flat surface. Touching each of the cubes takes one second. The sequence starts out simple, usually using two cubes, but becomes more complex until the subject’s performance suffers (Berch et al. 1998). Each stimulus item comprised a tapping pattern performed by the examiner, who pointed sequentially to a subgroup of the nine cubes. Participants were asked to copy the tapping pattern, which was indicated by the examiner in a backwards manner. The sequence complexity increased from one tap to nine taps at the highest level. The sequences were random, and the difficulty level was progressively raised by increasing the number of blocks tapped. There were three trials at each difficulty level. The subject’s spatial span was conventionally taken as the longest sequence in which at least two out of the three sequences were correctly reproduced. Participants with more correct responses had a better performance. Here, the backward spatial span was evaluated because it is more processing-intensive than the forward span and requires intense executive control mechanisms (engagement of the central executive WM sub-component in contrast to the VPT) and is also more sensitive to the age-dependent decline in spatial-sequential working memory (Kessels et al. 2008; Brown 2016). The exact instructions were as follows: “Look carefully at this wooden template. It has nine cubes pegged on it. I will touch n (starting from two) of the cubes and I would ask you to look at this carefully in order to touch the same cubes right after me in reverse order. Are you ready?”

### 2.5. State Affect

**Positive and Negative Affect Schedule (PANAS):** PANAS is a brief self-report questionnaire that was selected for the present experiment as it can reflect possible emotional reactions by measuring positive and negative affect (Watson and Clark 1999). The items of the PANAS are 10 adjectives for positive and 10 adjectives for negative affect: interested, distressed, excited, upset, strong, guilty, scared, hostile, enthusiastic, proud, irritable, alert, ashamed, inspired, nervous, determined, attentive, jittery, active, and afraid. Responses to different words describe feelings and emotions ‘right now’ at the time the individual completes the questionnaire (thus reflecting state, not trait). They were self-reported by the participants on a Likert-type scale ranging from 1 (very slightly, not at all) to 5 (extremely). The completion takes 5–10 min. The score range is 10–50 for both positive and negative affect. The validity of the PANAS instrument was demonstrated for the present sample by computing the Cronbach alpha coefficient in the silence condition, where the coefficient was acceptable for both the negative (α = 0.769) and positive affect items (α = 0.663).

### 2.6. Procedure and Measurable Parameters

All participants were tested in one session (for a total of two hours). At first, they filled out a demographic questionnaire, and then they were given a general oral explanation of the tasks that they were asked to perform later on, immediately after the listening conditions. Before the beginning of the main experiment, participants had the opportunity to adapt the volume of pre-recorded noise with verbal instructions to a level that would allow them to listen clearly. Participants were examined individually, as the two visuospatial tests required the presence of an examiner for each participant during the assessment.

Three different musical conditions were used, and silence served as a control condition. Participants were exposed consecutively to pieces by Mozart, Vivaldi, Glass, and silence. The conditions were randomized across participants with the use of the Latin square design. Between each of the four conditions, there was a short break. During the break, each participant performed the two cognitive tests (Corsi blocks backwards and the VPT). Thus, each participant had 4 measures for the first test and 4 measures for the second test. Half of the participants were examined first with the Corsi for all four music conditions and then with the VPT. The other half were given the same tests in reverse order. Data collection was conducted in a paper-and-pencil way. Additionally, the participants were asked to complete the PANAS by scoring the degree to which they experienced the conditions emotionally as positive or negative. Finally, at the end of the experiment, all participants were asked to indicate which of the four conditions was their favorite.

### 2.7. Statistical Analysis

Measures of the Corsi, VPT, and PANAS emotional state were subjected to analysis of variance with repeated measures (ANOVA) with two between-subjects variables: age(young vs. older participants) and excerpt preference with 4 levels (Mozart vs. Vivaldi vs. Glass vs. silence) and one within-subjects variable: music condition(MC) with 4 levels (Mozart vs. Vivaldi vs. Glass vs. silence). Because the order of tests was counterbalanced across subjects, factor order was not included in the general analysis. The Greenhouse–Geisser correction was applied to the within-subjects factors with more than two levels. Original *df* and corrected *p* values are reported. Simple effects were tested using the paired *t*-test, with the alpha level being set at *p* = 0.03 after correction for multiple testing of non-independent variables (Nyholt 2004; Derringer 2018). The effect size was controlled by computing the partial eta squared (*η_p_*²). Group distribution was evaluated by performing the chi-square (χ^2^) statistics.

## 3. Results

The distribution of participants according to preference for musical excerpts is presented in Table 1. The two age groups did not differ with respect to the distribution of musical preference (χ^2^(3, 310) = 2.35, *p* = 0.5).

### 3.1. Corsi Blocks Task

Age Effect. Young adults (as expected) manifested significantly higher Corsi scores in all conditions compared to older adults (Age, *F*(1, 301) = 203.7, *p* < 0.001, *η_p_*² = 0.400).

Preference Effect. Choice of music did not have a main effect on Corsi blocks backwards performance (*F*(3, 301) = 1.2, *p* > 0.3), and no interaction with Age was found (Age × Preference, *F*(3, 301) = 1.0, *p* > 0.3).

**Figure 1:**
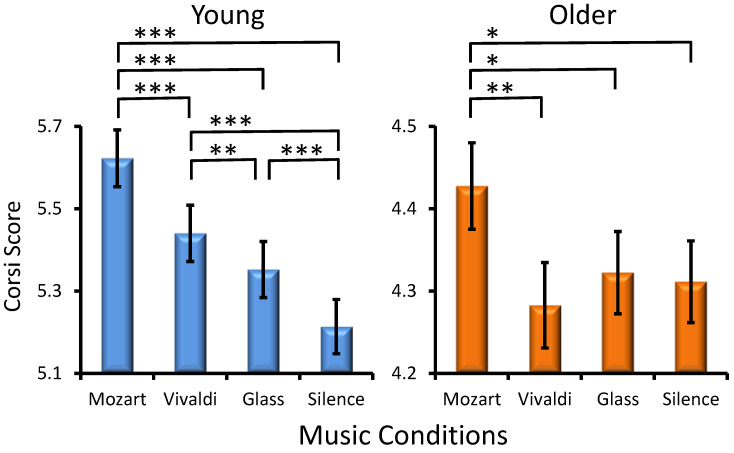
Effects of the music conditions on young and older adult performance in the Corsi Blocks Task. Significant differences between music conditions are illustrated for each age group: paired *t*-test, * *p* < 0.03, ** *p* < 0.01, *** *p* < 0.001.

Musical Condition Effect. A significant main influence was found for Musical Condition (*F*(3, 301) = 17.2, *p* < 0.001, *η_p_*^2^= 0.054), with all participants performing better after Mozart than after other excerpts (Figure 1). However, an interaction of Age × MC was also found (*F*(3, 301) = 6.8, *p* < 0.001, *η_p_*^2^ = 0.02), indicating that young adults had the highest performance after listening to Mozart as compared to the other three conditions and the lowest performance after silence as compared to all music conditions (Figure 1). Although older adults also manifested higher Corsi scores after Mozart, the difference between the other conditions was not significant. Significant differences between music conditions are depicted in Figure 1. No statistically significant interactions were found for Preference × Musical Condition (*F*(9, 301) = 0.86, *p* > 0.5) and Age × Preference × Musical condition (*F*(9, 301) = 1.1, *p* > 0.3).

### 3.2. Visual Patterns Test

Age Effect. Young adults manifested significantly higher VPT scores as compared to older adults (Age, *F*(1, 302) = 394.4, *p* < 0.001, *η_p_*² = 0.566).

Preference Effect. No main effect of Preference was found (*F*(3, 302) = 1.2, *p* > 0.3) for any of the age groups (Age × Preference, *F*(3, 302) = 1.7, *p* > 0.1).

**Figure 2:**
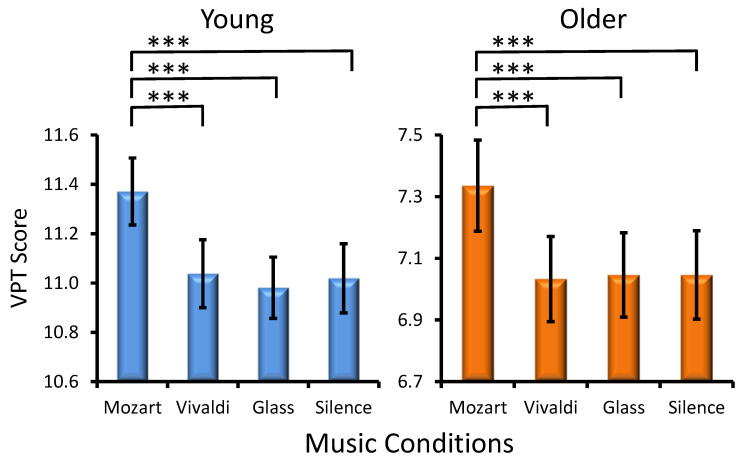
Effects of the music conditions on young and older adult performance in the Visual Patterns Test (VPT). Significant differences between music conditions are illustrated for each age group: paired *t*-test, *** *p* < 0.001.

Musical Condition Effect. A statistically significant main effect of MC was found (*F*(3, 906) = 18.2, *p* < 0.001, *η_p_*² = 0.088), again due to a prominent ‘Mozart effect’ in the two age groups (Age × Musical Condition, *F*(3, 302) = 0.5, *p* > 0.6). Significant differences between music conditions for each age group are shown in Figure 2. No significant interactions were yielded (Preference × Musical Condition, *F*(9, 302) = 1.1, *p* > 0.3, and Age × Preference × Musical Condition, *F*(9, 302) = 1.3, *p* > 0.2).

### 3.3. PANAS Positive Affect Subscale

**Figure 3:**
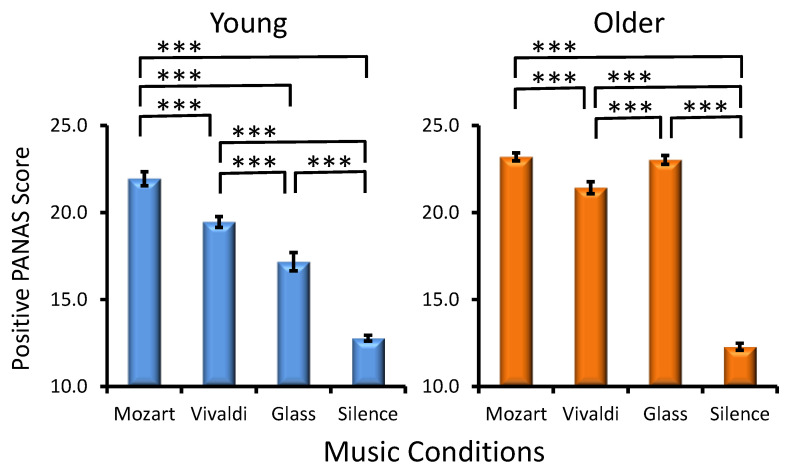
Effects of the music conditions on positive PANAS scores in young and older adults. Significant differences between music conditions are illustrated for each age group: paired *t*-test, *** *p* < 0.001.

Age Effect. As demonstrated in Figure 3, older adults manifested significantly higher PANAS positive scores in the majority of music conditions as compared to young adults (Age, *F*(1, 287) = 98.2, *p* < 0.001, *η_p_*² = 0.254).

Preference Effect. No main effect of Preference was found for the positive affect (*F*(3, 287) = 0.5, *p* > 0.6), but a statistically significant interaction effect was found for Age × Preference (*F*(3, 287) = 5.2, *p* = 0.002, *η_p_*² = 0.052).

Musical Condition Effect. A statistically significant main effect of MC was found (*F*(3, 861) = 288.9, *p* < 0.001, *η_p_*² = 0.500), reflecting the strongest positive affect after Mozart and the weakest positive affect after silence. As reflected by the significant Age × MC interaction (*F*(3, 861) = 25.7, *p* < 0.001, *η_p_*² = 0.082), although a significant Mozart effect existed in the two age groups, in young adults, positive affect differed between all other three conditions, in contrast to older adults, as seen in Figure 3, where statistically significant differences between music conditions are illustrated for each group. Preference × MC (*F*(9, 861) = 0.9, *p* > 0.5) and Age × Preference × MC interactions (*F*(9, 861)= 1.0, *p* > 0.4) were not significant.

### 3.4. PANAS Negative Affect Subscale

Age Effect. Young adults manifested overall higher PANAS negative scores as compared to older adults in the majority of music conditions (Age, *F*(1, 285) = 26.5, *p* < 0.001, *η_p_*² = 0.08).

Preference Effect. No main effect of Preference was found for the negative affect (*F*(3, 285) = 0.4, *p* > 0.7) in any age group (Age × Preference, *F*(3, 285) = 0.7, *p* > 0.5).

**Figure 4:**
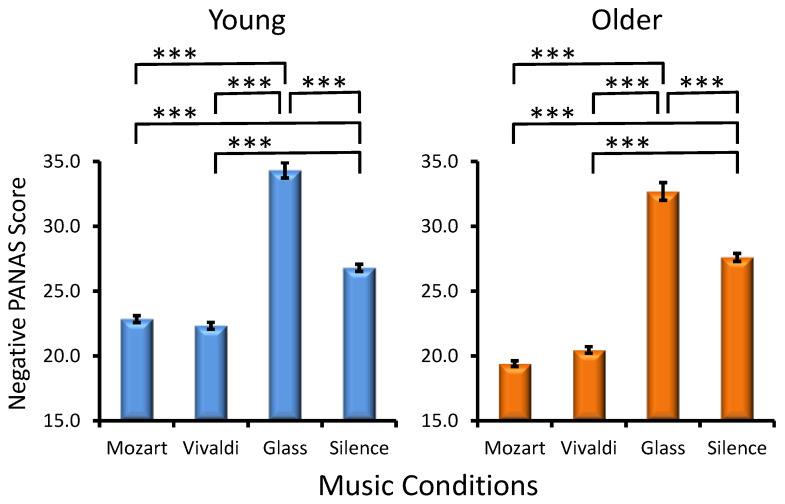
Effects of the music conditions on negative PANAS scores in young and older adults. Significant differences between music conditions are illustrated for each age group: paired *t*-test, *** *p* < 0.001.

Musical Condition Effect. A significant main effect for MC (*F*(1, 285) = 796.7, *p* < 0.001, *η_p_*² = 0.582) reflected the most expressed negative affect for Glass’s excerpt and the least expressed negative affect for Mozart’s music (Figure 4), with the latter effect being especially pronounced in older participants (Age × Condition, *F*(1, 285) = 33.2, *p* < 0.001, *η_p_*² = 0.030). No statistically significant interactions were found for MC × Preference (*F*(3, 285) = 1.0, *p* > 0.3) and Age × MC × Preference (*F*(3, 285) = 0.5, *p* > 0.7).

## 4. Discussion

The present study was designed to explore the effect of brief exposure to three musical pieces (Mozart, Vivaldi, and Glass) on visuospatial working memory in groups of young and older adults. Because the effect of these three excerpts has been previously tested in the same examinees on verbal working memory, with a critical influence of Vivaldi’s music being found (Giannouli et al. 2019), the major question was whether in the same design, the visuospatial WM sub-component would be affected in a different or similar way. Addressing this issue was relevant to highlight the possible mechanisms through which musical experience interacts with subsequent cognition—by involving circuit-specific (visuospatial or verbal) or unspecific executive control processes.

According to the major result, there was a prominent facilitating effect of the Mozart excerpt on visuospatial performance. Importantly, an enhancement of the visuospatial capacity after Mozart was observed (1) for the two tasks used in the present experiment, Corsi and VPT, independently of the differential involvement of central executive processes and different cognitive strategies in the two tasks (Baddeley 2000; Brown et al. 2006), and (2) in the two age groups, irrespective of the overall decline in the visuospatial capacity manifested by older subjects. More importantly, the improved visuospatial performance after Mozart was found in the comparisons with any other musical (Vivaldi and Glass) and nonmusical (silence) condition, whereas no reliable difference existed across the no-Mozart conditions. Despite the small effect size, these observations provide evidence for the functional specificity of the Mozart music in modulating the visuospatial sub-component of WM, particularly in the context of our previous study with the same musical excerpts and the same participants, where only the Vivaldi piece was efficient in modulating verbal memory.

This major result strongly supports the presence of a specific ‘Mozart effect’ on visuospatial processing that was originally described by Rauscher et al. (1993). The effect of Mozart music on promoting visuospatial cognition still remains debatable and controversial (Bottiroli et al. 2014). Since its discovery, it has been replicated in a variety of studies showing better performance in spatial reasoning after listening to Mozart (e.g., Smith et al. 2010; Rideout and Laubach 1996; Rideout et al. 1998; Jaušovec and Habe 2005; Jaušovec et al. 2006). A meta-analysis by Chabris (1999) including 20 publications on Mozart–silence comparisons has concluded that although the effect of Mozart music cannot be generalized or translated to different tasks, it is evident for a single task from the spatial–temporal processing domain. However, there are studies finding no significant differences in the visuospatial performance after listening to Mozart as compared to other musical or nonmusical conditions. Moreover, even if an enhancing effect of music was detected, it was not specific for Mozart (e.g., McCutcheon 2000; Lints and Gadbois 2003; Roth and Smith 2008; rev. Pauwels et al. 2014). On this background, the present results confirm that the Mozart effect does exist. Yet, as detailed below, its expression obviously depends on co-existing factors and influences.

In the present study, several additional tests were introduced in an attempt to clarify the role of these additional factors. First, with respect to the arousal hypothesis (Roth and Smith 2008), a silence condition was implemented here to be contrasted with music conditions. In the case of a major role for arousal in modulating subsequent cognition, significant differences would emerge for silence as compared with music stimulation, irrespective of the type of music. Critically, current results from the VPT demonstrate that visuospatial performance after silence did not differ from that after Vivaldi and Glass in any of the groups, and the same lack of silence effect was observed for the Corsi test in the older subjects. The silence effect was only detected for the Corsi test in young adults (Figure 1). The general pattern of these observations does not support the role of arousal in modulating the visuospatial sub-component of WM. Hence, the Mozart effect observed here cannot be considered in the context of the arousal framework.

Second, the possible contribution of emotional activation, which is essentially considered a major factor for the Mozart effect (Nantais and Schellenberg 1999; Panksepp and Bernatzky 2002; Thompson et al. 2001; Palmiero et al. 2016; Ribeiro et al. 2023),was accounted for in the present study. This was performed by assessing the effects of individual preference and the positive/negative effect induced by each musical piece. Although preference was expected to induce better performance (Getz et al. 2012), here no influence of music excerpt preference was found for any of the two visuospatial tests. Notably, the strongest positive affect and the weakest negative affect, as reflected by PANAS, were observed after Mozart in the two age groups (Figure 3 and Figure 4). In contrast, the negative affect was most expressed after Glass’s excerpt, whereas the positive affect was least expressed after silence. This pattern of results strongly implies that prevailing positive emotional reflections promote the enhancement of visuospatial processing after Mozart’s excerpt. Also, new evidence is provided that the negative effect is not as efficient as the positive one to modulate subsequent visuospatial performance. Together, these observations convincingly support the hypothesized critical role of the positive emotions for boosting the visuospatial WM sub-component (Thompson et al. 2001; Palmiero et al. 2016; Ribeiro et al. 2023). A possible explanation is that positive emotional reactions activate and prime the right hemisphere, resulting in improved spatial processing (Smith et al. 2010). In addition, neuroimaging techniques have demonstrated that listening to music consistently evokes activity in cortical and subcortical structures such as the amygdala, the nucleus accumbens, and the hippocampus responsible for emotion regulation (Koelsch 2018). Thus, neural networks commonly engaged in the processing of music and emotions may mediate the effects of emotional activations on cognition upon exposure to music and may have specifically contributed to the expression of the Mozart effect, as observed in the present study. Finally, emotional reactions induced by preceding music may have an indirect impact on the quality of cognition by improving the functioning of attentional systems in the brain (Fredrickson 1998; Eysenck et al. 2007).

An interesting finding here was the lack of connection between music preference and positive affect and cognition after listening. The present results revealed no association between music preference and performance on the two tests. This result may not be controversial because individual preference can be considered trait-dependent, whereas affect assessment may rather be driven by state-dependent factors. As such, individual preference may not be necessarily guided by an immediate positive emotional experience since it may be motivated by personality, disposition, temper, associations including implicit or explicit memories, memories of states (inspirational or melancholic), thoughts, etc. Also, «“affect” is an instinctual reaction to stimulation that occurs before the typical cognitive processes considered necessary for the formation of a more complex emotion(s)» (Zajonc 1980), which can also be misleadingly assessed (over- or under-stated) in a subjective instrument like PANAS (Daskalou and Sigkollitou 2012). Finally, such a disconnect may be explained by the activation of the autonomic nervous system by music appreciation (Pauwels et al. 2014), which can more directly and immediately influence the subjective assessment of affect.

Another major result of the present study reveals, however, that despite the influence of positive emotional activations on performance due to music appreciation, Mozart’s music has a separate, independent effect on the visuospatial WM sub-component. According to the observations, exposure to the Mozart excerpt produced a prominent, distinctive effect on VPT performance. Only after Mozart was the VPT performance significantly enhanced, and no differences among other conditions were found in either the young or older participants. For the Corsi task, in contrast, exposure to Vivaldi and Glass modulated visuospatial processing to a different extent and also produced an improvement relative to silence in young adults. Because the VPT is acknowledged to involve more directly visuospatial capacity and, to a lesser extent, non-specific processes of executive attention as compared to the Corsi task (Baddeley 2000), this observation is important in demonstrating that although the effect of Mozart music is modulated by emotional activations (as reflected by PANAS), there is an additional highly specific influence of Mozart on the visuospatial processing circuit. This conclusion is further supported by the results from the group of older participants. The older subjects, as expected (Rowe et al. 2008; Nicholls and English 2020), manifested an overall decline in visuospatial cognition in the two tasks. Nonetheless, a prominent, highly specific Mozart effect was detected only in the VPT in this age group, indicating a strong impact of Mozart’s music on the visuospatial circuit that was not modulated by attentional or cognitive capacity. Taken together, these observations contribute to the debate about whether the ‘Mozart effect’ exists and, if it does, how it affects cognitive performance. They provide new, strong evidence about the potential of Mozart music to specifically tackle processing within the visuospatial processing circuit, in addition to its capacity to enhance performance by evoking positive emotional activations.

How can this specificity of the Mozart excerpt on visuospatial processing circuits be explained? One neurophysiologic explanation is that listening to music primes those brain areas that are concerned with spatial reasoning (Mellet et al. 1996; Jenkins 2001). Neuroimaging research is in line with this possibility since it has been demonstrated that brain areas activated during music processing, including the prefrontal, temporal, and precuneus regions, overlap with those activated by spatial–temporal tasks (Mellet et al. 1996). A related explanation is that there are some unique musicological features of Mozart’s music that have the capacity to distinctively recruit visuospatial circuits. In the present study, the three musical excerpts were chosen to be similar in tempo and level of complexity and to have a high index of periodicity following repetitive structures and a tonal center (Giannouli et al. 2017). It has been reported that particular components of music appreciation involving rhythm, pitch, meter, and melody activate many different brain areas, with many interconnections and interactions of networks (Warren 1999; Patel 2003; Liégeois-Chauvel et al. 1998). For example, rhythm and pitch discrimination are processed mainly in the left hemisphere, whereas timbre and melody are processed mainly in the right hemisphere. It is interesting to note that in an attempt to determine some unique physical features of Mozart music, Hughes and Fino (2000) have performed a musicological analysis of a wide range of pieces by Mozart, Bach, Chopin, and 55 other composers. An interesting characteristic of Mozart’s music was the emphasis on the average power of particular notes, notably G3, C5, and B5. An even more distinctive characteristic of Mozart’s music was the long-term periodicity within the 10–60 s range. It was suggested that this long-term periodicity recruits and synchronizes the brain networks engaged in visuospatial processing, thus enhancing performance in such tasks (Hughes and Fino 2000). However, it still remains an open question if and how some particular physical features of music that may be strongly dominant in Mozart’s work may be responsible for the specific engagement of visuospatial neural circuits.

One limitation of the present study was the use of the same music excerpts and the same participants. This choice was, however, purposeful in order to have comparable conditions across the experiments testing the verbal and visuospatial sub-components of WM. Another limitation was the administration of only two visuospatial working memory tests that have not been thoroughly examined so far in this type of experimental condition (the Corsi Backwards Task and the VPT). This restriction stemmed from practical reasons since the short-term effects of music would be difficult to measure if a full neuropsychological battery were used. Finally, the whole sample consisted only of Greeks, which renders impossible cross-cultural comparisons as the perception of the chosen musical excerpts may differ in other cultural environments.

The findings of this research, emphasizing Mozart’s music influence on visual working memory performance, may assist in shaping future cognitive empowerment programs across the lifespan (in young and older adults). By overcoming the methodological and statistical limitations of previous experiments with small samples of only young participants who are examined once with a specific single test, we have investigated whether and how music may serve as a potential intervention. The study results support the application of brief music exposure as an easy, cheap, accessible, and not time-consuming way of improving verbal WM, visual WM, and emotional status, even temporarily. Music can also be an ideal intervention to maximize working memory performance and reduce negative emotions in people with special needs, but we still know little about the mechanisms of this influence, even in healthy participants. Such programs that do not require effort and do not require training of the individual or supervision from a healthcare expert are in high demand not only in real-life settings (nursing homes, university classes, etc.), but also in virtual-environment computer-based programs. Especially for older adults with cognitive deficits, such findings can be implemented in future intervention program designs.

## 5. Conclusions

The results of our present and previous studies demonstrate different roles of Mozart’s and Vivaldi’s music in the quality of visuospatial and verbal performance, indicating a separate involvement of the visuospatial sketchpad and the phonological loop of working memory. They thus contribute to the debate of whether music has the potential to affect distinct processes within working memory in an excerpt- or composer-specific manner. In addition to these specific effects, the present results suggest that emotional activation and central executive attention are essentially involved in modulating the influence of music on subsequent cognition.

## Figures and Tables

**Table 1:** Number and distribution of participants according to age and musical preferences.

	Mozart	Vivaldi	Glass	Silence	Total
Young	32 (20.5%)	63 (39.9%)	38 (24.1%)	25 (15.8%)	158
Older	27 (17.7%)	53 (34.9%)	48 (31.2%)	24 (15.8%)	152
All participants	59 (19.0%)	116 (37.4%)	86 (27.7%)	49 (15.8%)	310

## Data Availability

Data available upon request by the first author.
